# Prospective associations between depression and risk of hospitalisation for infection: Findings from the UK Biobank

**DOI:** 10.1016/j.bbi.2022.02.023

**Published:** 2022-05

**Authors:** Amy Ronaldson, Jorge Arias de la Torre, Rodica Sima, Mark Ashworth, David Armstrong, Ioannis Bakolis, Matthew Hotopf, Alexandru Dregan

**Affiliations:** a Institute of Psychiatry, Psychology and Neuroscience (IoPPN), King’s College London, London, UK; b University of Agricultural Sciences and Veterinary Medicine Cluj-Napoca, Faculty of Horticulture, Cluj-Napoca, Romania; c School of Life Course and Population Sciences, King’s College London, London, UK; d South London and Maudsley NHS Foundation Trust, London, United Kingdom

**Keywords:** Depression, Infection, Infectious disease, Hospitalisation, Inpatient, Antidepressants

## Abstract

**Background:**

Associations between depression and non-communicable disease have been well-described. However, the evidence for its role in the development of infectious disease is less understood. We aimed to examine prospective associations between depression and risk of hospitalisation for infection in middle-aged adults from the UK Biobank (linked with Hospital Episode Statistics) and assessed the role of several depression-related factors.

**Methods:**

We assessed prospective associations between depression status at the baseline assessment (2006–2010) and hospitalisations for infection up to the end of March 2016 in 460,418 middle-aged adults enrolled in the UK Biobank (mean age = 56.23 ± 8.11 years, 53.5% female). Cox regression was used to assess associations between depression and subsequent hospitalisations for any infections, as well as infection subtypes, viral infections, and bacterial infections. Amongst those with depression, we also examined the role of depression duration, the age of onset, and the use of antidepressants in hospitalisation risk.

**Results:**

Depression at baseline was prospectively associated with an increased risk of hospitalisation for infection (adjusted hazard ration (aHR) = 1.20, 95% confidence interval (CI) = 1.16 to 1.25). This association was found for all infection subtypes apart from infections of the central nervous system (*p* = 0.911) and the skin (*p* = 0.313). Receipt of a depression diagnosis in late adulthood and use of antidepressants (but only in those with none/mild depressive symptoms at baseline) increased the risk of hospitalisation for infection amongst those with depression.

**Conclusions:**

These findings suggest that depression might be a risk factor which could be used to identify those at risk of hospitalisation for infection. Future research is required to understand the underlying factors that might result in this increased risk, so that targeted interventions can be developed.

**Funding:**

AD and AR are funded by Guy’s Charity grant number EIC180702 (MLTC Challenge Fund); AD and JAT are co-funded by MRC and NIHR through grant number MR/S028188/1. IB is supported by the NIHR Maudsley BRC and by the NIHR Collaboration for Leadership in Applied Health Research and Care South Londnoo at King's College Hospital NHS Foundation Trust, King's College London. The views expressed are those of the author[s] and not necessarily those of the ESRC, NIHR, the Department of Health and Social Care or King’s College London.

## Highlights

• Depression is prospectively associated with an increased risk of hospitalisation for a wide range of infections, apart from infections of the central nervous system and skin.
• The life stage at which depression is diagnosed and use of antidepressants appear to play a role in increasing hospitalisation risk in those with depression.
• Future research needs to focus on understanding the underlying factors that might link depression and infection risk.

## 1 Introduction

In the UK, infectious disease accounts for approximately 7% of deaths and 8% of hospital bed days (UK Parliament: The Parliamentary Office of Science and Technology, 2017), with the total economic burden from infectious disease in England estimated to be more than £30 billion annually (Davies et al., 2013). Understanding the risk factors for contracting infectious disease and identifying the most vulnerable is key for targeted prevention. To date, research has largely focused on biological, social, and environmental indices, with rather less consideration being given to psychological factors. Psychological stress and depression are known to increase levels of systemic inflammation and suppress and/or dysregulate aspects of innate and adaptive immunity (Dhabhar, 2014, Osimo et al., 2019, Segerstrom and Miller, 2004). Therefore, it is plausible that these psychological factors might play a role in the development of infectious disease in people with depression.

Associations between depression and non-communicable disease have been well-described (Poole and Steptoe, 2018, Ronaldson et al., 2021). The development of depression as a sequela of infectious disease is also well-established, particularly following respiratory infection (Bornand et al., 2016, Mazza et al., 2020). However, the evidence for its role in the development of infectious disease is less understood. Longitudinal studies have shown that depression is prospectively associated with increased risk of acute respiratory tract infections (Falagas et al., 2010), herpes zoster virus (Irwin et al., 2011), intestinal *E. coli* infections (Mulugeta et al., 2020), pneumonia and meningitis (Seminog and Goldacre, 2013), and COVID-19 (Yang et al., 2020). Depression has also been found to raise the likelihood of post-operative infections (Cancienne et al., 2017) and infectious disease mortality (Davydow et al., 2016, Hamer et al., 2019). The largest and most comprehensive study to date looked at associations between depression and hospital contacts for infection and was carried out using data from linked Danish registries (Andersson et al., 2016). The authors found that depression was associated with an overall increased risk of severe infection, with the highest increase seen for sepsis. However, due to the nature of the data the authors were unable to adjust for several potential confounding factors such as socioeconomic status (SES), smoking, obesity, and physical health status. Moreover, mental health data captured in the Danish registries are solely based on diagnoses given in psychiatric settings meaning that underreporting of depression was highly likely.

Therefore, in the current study we examined prospective associations between depression and hospitalisations for infections using data from the UK Biobank which provides a more comprehensive measure of depression and allows for the adjustment of several relevant factors. More specifically, we sought to examine associations between depression and the risk of specific infection subtypes, including viral and bacterial infections. Amongst participants with depression, we assessed whether a number of depression-related factors associated with risk of hospitalisation for infectious disease, namely duration of depression, age of depression onset, and use of antidepressants.

## 2 Method

### 2.1 Study design and participants

The UK Biobank is a large population-based prospective study established for the investigation of the determinants of disease in middle- and older-aged adults. Data were collected from more than 500,000 participants aged between 40 and 69 years from 22 different assessment centres across the England, Scotland, and Wales between 2006 and 2010 (Sudlow et al., 2015). Participants had to be registered with a general practitioner (GP) and live within 25 miles of an assessment centre to take part. Detailed accounts of sociodemographic, lifestyle, and medical information were gathered from all patients recruited to the study using a touchscreen questionnaire during the baseline assessment. Patients also provided information about medical diagnoses in a computer-assisted personal interview administered by trained interviewers. Hospital Episode Statistics (HES) have been linked to the UK Biobank. HES are national data for England that contain ICD-10 diagnoses for hospital inpatient admissions. All participants gave informed consent. The UK Biobank has ethical approval from the NHS National Research Ethics Service (16/NW/0274).

In the current study, the sample were selected based on those who completed the baseline assessment (N = 502,599). Participants were excluded from the analysis if they had suspected immunosuppression at any time defined as a diagnosis of HIV/AIDS (self-report and/or ICD-10 codes: B20-24) and/or a malignant neoplasm (self-report and/or ICD-10 codes: C00-C97 with the exception of non-melanoma skin cancer C44) (Andersson et al., 2016). The sample were followed from the baseline assessment until the end of the study period (March 2016), leading to a follow-up period of 6–10 years.

### 2.2 Depression

This study used multiple sources to determine depression at baseline: self-report, the Patient Health Questionnaire (PHQ)-2 (depression items) (Kroenke et al., 2003), and linked HES. A detailed description of how baseline depression was measured in the current study is provided in previous work assessing depression in the UK Biobank (Dregan et al., 2020). The use of different data sources provides a more comprehensive definition of depression, a heterogenous condition, than is possible with any of the individual approaches. While PHQ-2 scores may capture early depressive symptoms, HES data likely encompasses severe depression. In this way, our definition of depression provides a more detailed conceptualisation of depression ranging from mild depressive symptoms to clinical depression.

A subsample of participants who self-reported depression at the baseline assessment also provided the year and/or the age they received their diagnosis. In the current study, year of diagnosis was subtracted from the year of assessment to create a duration of depression variable (years). Age at diagnosis was used to create a categorical variable where participants received their diagnosis in adolescence/early adulthood (10–39 years), middle age (40–59 years), and late adulthood (60 + years)(Medley, 1980). At the baseline assessment, participants were asked whether they were taking any regular medication and a nurse interviewer took the names of the medications taken. A pre-existing code list of antidepressants was used to extract these data (Davis et al., 2019). In the current study we created a binary antidepressant usage variable (yes/no) for those with depression at baseline. In order to account for depression severity, we used PHQ-2 (depression) scores to separate participants into those likely to have moderate/severe depressive symptoms (PHQ-2 score ≥ 2) and none/mild depressive symptoms (PHQ-2 score < 2) at the baseline assessment (Arroll et al., 2010).

### 2.3 Hospitalisation for infections

Linked hospital admission records (HES) were used to identify a primary or secondary diagnosis of infection. The following ICD-10 codes were used to identify central nervous system (CNS) infections (A17, A80-A81, A85-A89, B00.3-B00.4, B01.0-B01.1, B02.0-B02.2, B05.0-B05.1, B06.0, B2.61-B26.2, G00-G01, G02.0, G03, G04.2, G05.0-G05.1, e.g., meningitis, viral encephalitis), gastrointestinal infections (A00-A05, A08, e.g., salmonella, shigellosis), liver infections (B15-B19, e.g., hepatitis A), respiratory infections (A15-A16, A36-A38, J00-J06, J09-J18, J20-J22, e.g., pneumonia, laryngitis), sepsis (A40-A41, e.g., streptococcal sepsis), skin infections (A46, B00-B09, L00-L05, L08, e.g., cellulitis, measles), urogenital infections (N30.0, N39.0, N41.0–41.1, N71-N72, e.g., cystitis, prostatitis), and other infections (A18-A19, A31-A32, A39, A42-A44, A48-A49, B25-B27, B30, B33-B34, B95-B98, H62.0-H62.1, H67.0-H67.1, M00, M01.0-M01.5, N61, e.g., bone infection, mastitis)(Andersson et al., 2016). Diagnoses were looked at altogether (‘any infection’), by subtype, and were also categorised into bacterial and viral infection subgroups (see Table S1, S2, and S3 in Supplementary material for a detailed list of infection types).

### 2.4 Covariates

Several covariates were included, and their selection was informed by previous literature and theory. Sociodemographic variables included age, sex, and social deprivation which was measured at the baseline assessment. Social deprivation was based on the Townsend deprivation indices (Townsend, 1987) derived from aggregated data on car ownership, household overcrowding, owner occupation, and unemployment. Higher scores were indicative of higher deprivation. Being a current or past smoker (yes/no), the frequency of alcohol intake, and body mass index (BMI, kg/m^2^) measured at the baseline assessment were also included as covariates. Physical long-term conditions (LTC) were measured at the baseline assessment using both self-report lifetime diagnoses and linked HES. A total of 36 LTCs were used in the current study to assess physical LTC count (e.g., asthma, cancer, diabetes). A detailed description of these conditions are provided in previous work based on UK Biobank data (Ronaldson et al., 2021). Serum C-reactive protein (CRP) levels (mg/L) from blood samples taken at the baseline assessment were measured by immunoturbidimetric high-sensitivity analysis on a Beckman Coulter AU5800 (UK Biobank, n.d.). CRP is a marker of inflammation and infection (Sproston and Ashworth, 2018) and elevated levels of CRP are associated with increased risk of depression (Wium-Andersen et al., 2013).

In analyses relating to liver infections, levels of alanine aminotransferase (ALT) were included as an additional covariate. ALT is a commonly used biomarker for liver damage (Goorden et al., 2013) and levels were measured (U/L) from blood samples taken at the baseline assessment using the enzymatic rate method (Beckman Coulter AU5800) (UK Biobank, n.d.).

### 2.5 Statistical analyses

Variables were summarised as means and standard deviations, and frequencies. Comparisons between those with and without depression at the baseline assessment were performed using Mann-Whitney U and chi squared tests. Cox proportional hazards regressions were used to compute hazard ratios with accompanying 95% confidence intervals (CI) to summarise unadjusted, age- and sex-adjusted, and fully adjusted prospective associations between depression, and depression-related factors, and risk of hospitalisation for infectious disease. The proportional hazards assumption was assessed by testing Schoenfeld residuals (Grambsch and Therneau, 1994). In all analyses, the outcome was considered to be the first incidence of hospital admission for an infection over the study period, taking into account censored data points. The timescale was calendar time (days). In fully adjusted models we controlled for *a priori* confounders including age, sex, social deprivation, BMI, smoking status, frequency of alcohol intake, number of physical LTCs, and CRP levels. As CRP data were clearly skewed, they were log-transformed and the geometric means and associated confidence intervals (CI) were calculated. Baseline ALT levels were included in the analysis pertaining to hospital admissions for/with liver infections.

Data were missing for several variables: social deprivation (0.1%), BMI (0.6%), frequency of alcohol intake (0.3%), CRP (6.4%), ALT level (6.6%). As multivariable normality could not be assumed, multiple imputation using chained equations with 10 imputations was performed to deal with missing data (Azur et al., 2011). Multiple imputation included outcome and exposure variables as well as covariates in order to account for the complex interrelationships between all study variables. All fully adjusted analyses were based on imputed data.

All analyses were conducted in STATA 15.1 (Stata Corp LLP, College Station, TX).

### 2.6 Sensitivity analysis

It is possible that patients may have died from reasons other than infectious disease before experiencing a hospitalisation for infectious disease. Data from the UK Biobank baseline assessment centre were linked to national mortality records by UK Biobank. Therefore, we performed a competing risk analysis examining fully adjusted associations between depression and hospitalisation for infectious disease, with the competing outcome being death from reasons other than infectious disease.

It is also possible that depressive symptoms measured at the baseline assessment are as a result of persistent infection prior to baseline (Bornand et al., 2016, Mazza et al., 2020). Therefore, we examined associations between depression and hospitalisation for infectious disease excluding hospitalisations that took place in the 12 months following the baseline assessment.

We repeated the analysis using non-imputed data to examine whether the imputed dataset generated similar results.

## 3 Results

### 3.1 Sample characteristics

**Fig. 1:**
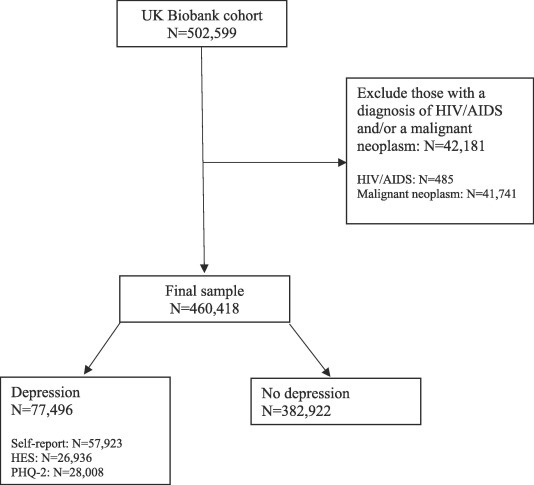
Flow diagram of sample selection.

The overall sample comprised those who completed the baseline UK Biobank assessment without suspected immunosuppression at any time defined as a diagnosis of HIV/AIDS and/or a malignant neoplasm (n = 460,418). A sample selection flowchart is provided in Fig. 1. Table 1 describes baseline characteristics for the sample by depression status. At baseline, 16.8% (n = 77,496) of participants were considered to have depression. Those with depression were younger (depression: 55.14 ± 8.09 years, no depression: 56.46 ± 8.10, *p* < 0.001), more likely to be female (depression: 58.8%, no depression: 52.4%, p < 0.001), and had higher levels of social deprivation (depression: −0.42 ± 3.44, no depression: −1.46 ± 2.99, *p* < 0.001). 15.5% of people with depression were current smokers at baseline compared with 9.7% of non-depressed and were more likely to abstain from drinking alcohol (depression: 13.3%, no depression: 7%). Those with depression had higher BMI values (depression: 28.16 ± 5.37, no depression: 27.30 ± 4.64, *p* < 0.001). The number of physical LTCs was higher in depressed participants (1.46 ± 1.45) compared to the non-depressed (1.10 ± 1.20) (*p* < 0.001). CRP levels were also higher in those with depression (0.77, 95% CI 0.77 to 0.78) compared to those without (0.68, 95% CI 0.67 to 0.68) (*p* < 0.001), as were ALT levels (depression: 23.99 ± 14.90, no depression: 23.52 ± 13.58, *p* = 0.012).

**Table 1:** Sample characteristics (n = 460,418).

	Depression (77,496, 16.8%)	No depression (382,922, 83.2%)	*p* value
	*M ± SD, N(%)*	*M ± SD, N(%)*	
Age	55.14 ± 8.09	56.46 ± 8.10	**<0.001**
Female	45,594 (58.8)	200,848 (52.4)	**<0.001**
Social deprivation*	−0.42 ± 3.44	−1.46 ± 2.99	**<0.001**
BMI (kg/m^2^)	28.16 ± 5.37	27.30 ± 4.64	**<0.001**
Smoking status			**<0.001**
*Never smoked*	41,336 (53.3)	213,040 (55.6)	
*Past smoker*	24,163 (31.2)	132,815 (34.7)	
*Current smoker*	11,997 (15.5)	37,067 (9.7)	
Alcohol intake			**<0.001**
*Daily/almost daily*	12,723 (16.4)	80,233 (20.9)	
*3 or 4 times a week*	13,513 (17.4)	93,038 (24.3)	
*Once or twice a week*	18,867 (24.3)	100,576 (26.3)	
*1 to 3 times a month*	9632 (12.4)	42,213 (11.0)	
*Special occasions only*	12,463 (16.1)	40,202 (10.5)	
*Never*	10,296 (13.3)	26,660 (7.0)	
Number of physical LTCs	1.46 ± 1.45	1.10 ± 1.20	**<0.001**
CRP (mg/L)**	0.77, 0.77 to 0.78	0.68, 0.67 to 0.68	**<0.001**
ALT level (U/L)	23.99 ± 14.90	23.52 ± 13.58	**0.012**
Hospitalisations for infection			
Any infection	4156 (5.4)	14,006 (3.7)	**<0.001**
*CNS*	38 (0.05)	181 (0.05)	0.837
*GI*	300 (0.4)	813 (0.2)	**<0.001**
*Liver*	40 (0.05)	77 (0.02)	**<0.001**
*Respiratory*	1834 (2.4)	5592 (1.5)	**<0.001**
*Sepsis*	312 (0.4)	1175 (0.3)	**<0.001**
*Skin*	880 (1.1)	3312 (0.9)	**<0.001**
*Urogenital*	987 (1.3)	3304 (0.9)	**<0.001**
*Other*	246 (0.3)	789 (0.2)	**<0.001**
Viral infection	1216 (1.6)	3732 (1.0)	**<0.001**
Bacterial infection	2314 (3.0)	8224 (2.1)	**<0.001**

ALT = Alanine aminotransferase; BMI = Body mass index; CNS = Central nervous system; CRP = C-reactive protein; GI = Gastrointestinal; LTC = Long-term condition

*Social deprivation was measured using the Townsend Deprivation Index. Positive values indicate areas of high material deprivation, whereas negative values indicate relative affluence

**The geometric mean and 95% confidence interval is reported for log-transformed CRP levels

### 3.2 Prospective associations between depression and risk of hospitalisation for infection

Prospective associations between baseline depression and subsequent risk of hospitalisation for infection are presented in Table 2. After adjusting for *a priori* covariates, Cox regression revealed that depression was prospectively associated with an increased likelihood of hospitalisation for any infection (adjusted hazard ratio (aHR) = 1.20, 95% CI = 1.16 to 1.25). When we looked at infection subtypes, we found that depression was associated with an increased likelihood of hospitalisation for all infection subtypes apart from CNS (*p* = 0.911) and skin (*p* = 0.313) infections. Depression was prospectively associated most strongly with hospitalisation for liver infections (aHR = 1.89, 95% CI = 1.26 to 2.85), followed by gastrointestinal (aHR = 1.41, 95% CI = 1.23 to 1.62), respiratory (aHR = 1.28, 95% CI = 1.21 to 1.35), urogenital (aHR = 1.26, 95% CI = 1.17 to 1.36), other (aHR = 1.19, 95% CI = 1.03 to 1.38), and sepsis (aHR = 1.19, 95% CI = 1.05 to 1.36) infections. Baseline depression was associated with increased risk of hospitalisation for both viral (aHR = 1.27, 95% CI = 1.18 to 1.36) and bacterial (aHR = 1.15, 95% CI = 1.10 to 1.22) infections with a stronger association seen for viral infections. Person-years of observation for each infection outcome ranged from 3,083,735 to 3,270,206.

**Table 2:** Prospective associations between depression and hospitalisation for infection.

	Unadjusted		Age and sex-adjusted		Fully adjusted*	
	*HR (95% CI)*	*p value*	*HR (95% CI)*	*p value*	*HR (95% CI)*	*p value*
Any infection	1.44 (1.39 to 1.49)	**<0.001**	1.54 (1.48 to 1.60)	**<0.001**	1.20 (1.16 to 1.25)	**<0.001**
Infection subtype						
*CNS*	1.06 (0.74 to 1.50)	0.760	1.10 (0.77 to 1.56)	0.592	0.98 (0.68 to 1.41)	0.911
*GI*	1.83 (1.60 to 2.09)	**<0.001**	1.91 (1.67 to 2.19)	**<0.001**	1.41 (1.23 to 1.62)	**<0.001**
*Liver*	2.72 (1.84 to 4.02)	**<0.001**	2.66 (1.79 to 3.93)	**<0.001**	1.89 (1.26 to 2.85)	**0.002**
*Respiratory*	1.60 (1.52 to 1.69)	**<0.001**	1.75 (1.66 to 1.85)	**<0.001**	1.28 (1.21 to 1.35)	**<0.001**
*Sepsis*	1.32 (1.16 to 1.49)	**<0.001**	1.44 (1.27 to 1.63)	**<0.001**	1.19 (1.05 to 1.36)	**0.008**
*Skin*	1.30 (1.21 to 1.41)	**<0.001**	1.36 (1.26 to 1.47)	**<0.001**	1.04 (0.96 to 1.12)	0.313
*Urogenital*	1.49 (1.39 to 1.60)	**<0.001**	1.61 (1.50 to 1.73)	**<0.001**	1.26 (1.17 to 1.36)	**<0.001**
*Other*	1.55 (1.34 to 1.79)	**<0.001**	1.54 (1.34 to 1.78)	**<0.001**	1.19 (1.03 to 1.38)	**0.019**
Viral infection	1.60 (1.49 to 1.71)	**<0.001**	1.67 (1.56 to 1.79)	**<0.001**	1.27 (1.18 to 1.36)	**<0.001**
Bacterial infection	1.39 (1.32 to 1.45)	**<0.001**	1.48 (1.41 to 1.55)	**<0.001**	1.15 (1.10 to 1.22)	**<0.001**

CNS = Central nervous system; GI = Gastrointestinal

*Covariates: Age, sex, social deprivation, BMI, smoking status, frequency of alcohol intake, number of long-term physical conditions, CRP level. ALT level was also adjusted for in the analysis where hospital admissions for liver infection were the outcome.

### 3.3 Depression-related factors and risk of hospitalisation for infection

Prospective associations between duration of depression, age of onset, and antidepressant use at baseline amongst participants with depression are presented in Table 3. Duration of depression was estimated in a subsample of participants with depression who provided the year of diagnosis at the baseline assessment (n = 27,483). The average number of years since initial receipt of a depression diagnosis was 24.84 (SD = 16.23). In the fully adjusted model, duration of depression was not associated with risk of hospitalisation for infection (*p* = 0.995). At the baseline assessment, 21,220 participants provided the age of onset of depression. 57.9% (n = 12,296) received their depression diagnosis in adolescence/early adulthood, 38.2% (n = 8105) in middle age, and 3.9% (n = 819) in late adulthood. Compared to those diagnosed in middle age, those diagnosed in late adulthood had an increased risk of hospitalisation for infectious disease (aHR = 1.41, 95% CI = 1.05 to 1.89). Those who were diagnosed with depression in adolescence/early adulthood did not differ from those diagnosed in middle age (*p* = 0.273).

**Table 3:** Associations between depression duration, age of onset, and use of antidepressants and hospitalisation for any infection in participants with depression.

		Unadjusted		Age and sex-adjusted		Fully adjusted*	
	*Sample size*	*HR (95% CI)*	*p value*	*HR (95% CI)*	*p value*	*HR (95% CI)*	*p value*
Depression duration (years)	27,483	1.01 (1.00 to 1.01)	**<0.001**	1.01 (1.00 to 1.01)	**0.003**	1.00 (0.99 to 1.00)	0.995
Age of onset of depression	21,220						
*Middle-age*		Ref		Ref		Ref	
*Adolescence/early adulthood*		1.14 (1.01 to 1.30)	**0.038**	1.24 (1.09 to 1.41)	**0.001**	1.08 (0.94 t0 1.23)	0.273
*Late adulthood*		1.40 (1.05 to 1.86)	**0.022**	1.14 (0.85 to 1.53)	0.370	1.41 (1.05 to 1.89)	**0.023**
Use of antidepressants							
*Moderate/severe symptoms*	35,069	1.37 (1.24 to 1.51)	**<0.001**	1.38 (1.25 to 1.52)	**<0.001**	1.10 (0.99 to 1.21)	0.064
*None/mild symptoms*	42,427	1.31 (1.18 to 1.47)	**<0.001**	1.33 (1.19 to 1.49)	**<0.001**	1.15 (1.02 to 1.29)	**0.017**

*Covariates: Age, sex, social deprivation, BMI, smoking status, frequency of alcohol intake, number of long-term physical conditions, CRP levels.

Among those with a history of depression, PHQ-2 scores indicated that 45.2% (n = 35,069) of people had moderate/severe depressive symptoms at the baseline assessment. Reported use of antidepressant medication at the baseline assessment (overall: n = 17,742, 22.9%, moderate/severe depressive symptoms: n = 9787, 27.9%; none/mild depressive symptoms: n = 7955, 18.7%) was prospectively associated with an increased risk of hospitalisation for infection in those with none/mild depressive symptoms (aHR = 1.15, 95% CI = 1.02 to 1.29) but not for those with moderate/severe depressive symptom (*p* = 0.064).

### 3.4 Sensitivity analysis

Results from a competing risk analysis looking at associations between depression and hospitalisation for infectious disease with death from causes other than infectious disease included as the competing outcome are presented in Table S4 (Supplementary material). Results were similar to those obtained using Cox proportional hazards regression models.

We examined associations between depression status and hospitalisations for infections that occurred from 12 months after the baseline assessment. Results are presented in Table S5 (Supplementary Material). Results were similar to the main analysis with the exception of the association between depression and hospitalisation for other infection subtypes which became borderline (aHR = 1.17, 95% CI = 1.00 to 1.37).

Results from the analysis repeated on unimputed data are presented in Table S6 in the Supplementary Material and are very similar to the results from the imputed analysis. However, associations between depression and hospitalisation with/for sepsis and Other infections became non-significant.

Schoenfeld residuals indicated that proportional hazard assumptions had been violated for some variables. However, this is not surprising in a dataset of this size seeing as these tests rely on failure to reject the null hypothesis, the likelihood of which decreases as sample size increases. Therefore, we performed a post-hoc sensitivity analysis where associations between depression and hospitalisations were modelled using logistic regression where time-to-event is not considered. Results are presented in Table S7 (Supplementary material) and were similar to those obtained using Cox proportional hazards regression models.

## 4 Discussion

In a large population of middle-aged adults, a history of depression was prospectively associated with an increased risk of hospitalisation for infection, even after adjusting for a range of relevant covariates. When we looked at infection subtypes, depression was associated with increased risk of hospitalisation for liver, gastrointestinal, respiratory, urogenital, sepsis, and ‘other’ infections, but not with CNS and skin infections. Risk of hospitalisation for viral and bacterial infections was increased in those with a history of depression, and this appeared to be stronger for viral infection.

The results from the current study are in keeping with previous longitudinal large-scale studies which have found depression to be associated with infectious disease incidence and mortality (Andersson et al., 2016, Davydow et al., 2016, Hamer et al., 2019). Furthermore, Andersson et al., also found depression to be unrelated to CNS infection incidence (Andersson et al., 2016). However, counter to our results, they found that depression was associated with incidence of more broadly defined skin infections (e.g., inpatient, outpatient, emergency admissions). Our definition of infection was restricted to hospitalisation, thus, likely to only capture the most severe cases of infection. We found that depression was more strongly associated with hospitalisation for viral infection than bacterial infection, which is in line with Hamer and colleagues who report stronger associations between psychological distress and viral infection mortality than bacterial infection mortality (Hamer et al., 2019). Andersson and colleagues report similar associations between depression and bacterial and viral infection incidence (Andersson et al., 2016).

Although not assessed here, there are multiple mechanisms through which depression could increase the likelihood of infection, and subsequent hospitalisations. First, depression might impact infection risk through changes in immune function. Dysregulation of neuroendocrine pathways (e.g., the hypothalamic–pituitaryadrenal axis) brought about by depression can lead to immunosuppression in the form of altered immune cell distribution, decreased lymphocyte proliferation, reduced virus-specific T-cell responses, and reduced memory T-cell responses, thereby increasing the risk of contracting infectious disease (Irwin and Miller, 2007). Moreover, there is growing evidence that there are bidirectional associations between depression and changes in the gut microbiome (Peirce and Alviña, 2019). The gut microbiome is known to interact with the immune system meaning that depression-induced changes in the microbiome may have implications for the spread of infection (Zheng et al., 2020). Depression might also increase risk of infection through unhealthy behaviours. Depression is associated with decreased physical activity (Roshanaei-Moghaddam et al., 2009) and poor sleep quality (Alvaro et al., 2013) which have both been shown to impact immune function and increase infection risk (Irwin, 2012, Nieman and Wentz, 2019). In the current study, the outcome was hospitalisation for infectious disease. It is possible that other depression-related factors might increase the likelihood of hospitalisation regardless of infection severity, such as poor coping skills, lack of social support, and functional impairment (Greenglass et al., 2006).

Among a subsample of participants with depression, we found that those who received a depression diagnosis in late adulthood were more likely to be hospitalised compared to those who were diagnosed in middle age. Late life depression is known to be different to depression diagnosed earlier in the lifecourse (Blazer, 2003). Depression in late life is frequently comorbid with other psychiatric and physical conditions and is a common sequela of a number of ageing-related conditions such as coronary heart disease, stroke, and diabetes (Poole and Steptoe, 2018). What this implies is that depression in late life might be a proxy for poor physical health which could leave an individual prone to infection. Although we adjusted for a considerable number of physical conditions in the current study, it was not possible to account for the severity of these conditions and associated functional impairment which would certainly have implications for infection risk.

In the current study, we found that amongst those with a history of depression, use of antidepressants was associated with an increased risk of hospitalisation for infection. However, this association was only found in those with none/mild depressive symptoms at the UK Biobank baseline assessment. There is some evidence to suggest that the use of antidepressants is associated with increased risk of certain types of infection (Mikocka-Walus, 2013, Rogers et al., 2013). It might be that this increased risk is dependent on depression severity. In those with moderate to severe depression, increased risk of infection might be brought about through behavioural and biological changes. In those with less severe depression, it might be that use of antidepressants increases risk of some infections. Moreover, in patients with none/mild symptoms of depression, antidepressants might be prescribed for other reasons, such as neuropathic or chronic pain. Therefore, it could be that antidepressant use is an indicator of poor physical health or the presence of more symptomatic (i.e., painful) physical conditions which could be driving the risk for hospitalisation for infection. More work is needed to understand the role of antidepressants in infection risk.

The results of this study provide further evidence for the role of depression in infectious disease, suggesting that depression is a salient risk factor. The current COVID-19 pandemic highlights the need for identifying those at increased risk of infectious disease, and increased risk of hospitalisation for infectious disease. Detecting the most vulnerable is key for prevention and might have important public health implications such as informing targeted immunisation and the order of priority for vaccinations. A recent *Lancet Psychiatry* Commission called for more work to identify the underlying factors that might result in increased infection risk in those with common mental health disorders so that targeted interventions can be developed. Future research needs to unpick the clinical, biological, behavioural, and psychosocial factors that might link depression with increased infection risk, and increased risk of hospitalisation for infection.

### 4.1 Strengths and weaknesses

In addition to the large sample size, data integration in the UK Biobank from multiple sources allowed for a comprehensive definition of depression caseness at the baseline assessment. Linked hospital episode data provided detailed information about hospitalisations for infection. The UK Biobank also allowed us to adjust for a considerable number of relevant factors known to associate with both depression and risk of hospitalisation for infectious disease, which previous studies have been unable to do.

Several limitations need consideration. A major limitation associated with observational studies is difficulty establishing causality and residual confounding. Although we controlled for a considerable number of relevant confounders, we cannot reject the possibility of unmeasured confounders that might bias our results. Depression caseness was only measured once and therefore we have no way of accounting for the number of depressive episodes a person has experienced which is known to be a useful indicator of infection risk (Andersson et al., 2016). However, we did not find any association between depression duration and risk of hospitalisation for infection. Moreover, it is entirely possible that non-depressed participants at baseline went on to develop depression over the study period which we were unable to account for. However, post-baseline depression events would have limited impact on the robustness of baseline effect-sizes and act mainly as intermediate outcomes or mediators. In the current study, we used both primary and secondary ICD-10 codes to identify diagnoses of infection. This was to ensure that sample sizes were adequate to assess associations with depression and infection outcomes. However, it means that we cannot unpick whether infection was a reason for hospitalisation or acquired whilst in hospital. Among those with depression, age of onset of depression and antidepressant usage were gathered via self-report which are known to be subject to bias. Further, our measure of depression severity might have been insensitive leading to misclassification bias, where moderate to severe cases were wrongly classified as mild (and *vice versa*). The UK Biobank comprises middle-aged participants resident in the UK meaning that the generalisability of the results is limited to this population subgroup. The UK Biobank cohort is also known to differ from the general UK population in terms of demographic (more female, less deprived) and health (less smoking, lower alcohol intake, fewer self-reported health conditions) factors which will also affect the generalizability of results (Fry et al., 2017). However, more comprehensive UK data may have elicited stronger associations between depression and risk of hospitalisation for infection seeing as rates of depression and infection might have been higher in a more representative sample.

## 5 Conclusions and implications

The current study provided more evidence for the role of depression in infectious disease, showing that depression was prospectively associated with increased risk of hospitalisation for infection. This association was present for all infection subtypes apart from infections of the CNS or the skin, implying system-specific vulnerability. These findings suggest that depression might be a risk factor which could be used to identify those at risk of hospitalisation for infection. Future research is required to understand the underlying factors that might result in this increased risk, so that targeted interventions can be developed.

### 5.1 Contributors

AR was responsible for the conceptualisation, formal analysis, and writing of the original draft of the manuscript. JAT, RS, MA, DA, MH, IB, and AD contributed to the writing (review and editing) of the manuscript. AD was also responsible for data curation and funding acquisition.

### 5.2 Data sharing

Data from UK Biobank are available to all researchers upon making an application. The study was conducted under UK Biobank project number 34554.

## Declaration of Competing Interest

This study represents independent research part-funded by the National Institute for Health Research (NIHR) Biomedical Research Centre at South London and Maudsley NHS Foundation Trust and King’s College London. The views expressed are those of the author(s) and not necessarily those of the NHS, the NIHR or the Department of Health and Social Care. AR, JAT, RS, MA, DA, IB, MH, and AD have nothing to disclose.


