# Supplementary material for: Prospective associations between depression and risk of hospitalisation for infection: Findings from the UK Biobank
Source: Brain Behav Immun. 2022 May;102:292–8. doi: 10.1016/j.bbi.2022.02.023 (PMC11924240; doi:10.1016/j.bbi.2022.02.023)
Supplement: Supplementary data 1 [file mmc1.docx]

**Supplementary material**

| Table S1. List of infection subtypes by ICD-10 code | |
| --- | --- |
| **Infection type** | **ICD-10 code** |
| *Central nervous system infections* | |
| Tuberculous meningitis | A17 |
| Acute poliomyelitis | A80 |
| Atypical virus infections of central nervous system | A81 |
| Other viral encephalitis, not elsewhere classified (NEC) | A85 |
| Unspecified viral encephalitis | A86 |
| Viral meningitis | A87 |
| Other viral infections of central nervous system, NEC | A88 |
| Unspecified viral infection of central nervous system | A89 |
| Herpesviral meningitis | B00.3 |
| Herpesviral encephalitis | B00.4 |
| Varicella meningitis | B01.0 |
| Varicella encephalitis, myelitis and encephalomyelitis | B01.1 |
| Zoster encephalitis | B02.0 |
| Zoster meningitis | B02.1 |
| Zoster with other nervous system involvement | B02.2 |
| Measles complicated by encephalitis | B05.0 |
| Measles complicated by meningitis | B05.1 |
| Rubella with neurological complications | B06.0 |
| Mumps meningitis | B26.1 |
| Mumps encephalitis | B26.2 |
| Bacterial meningitis, not elsewhere classified | G00 |
| Meningitis in bacterial diseases classified elsewhere | G01 |
| Meningitis due to other and unspecified causes | G03 |
| Meningitis in viral diseases classified elsewhere | G02.0 |
| Bacterial meningoencephalitis and meningomyelitis, NEC | G04.2 |
| Encephalitis, myelitis, and encephalomyelitis in bacterial diseases classified elsewhere | G05.0 |
| Encephalitis, myelitis, and encephalomyelitis in viral diseases classified elsewhere | G05.1 |
| *Gastrointestinal infections* | |
| Cholera | A00 |
| Typhoid and paratyphoid fevers | A01 |
| Other salmonella infections | A02 |
| Shigellosis | A03 |
| Other bacterial intestinal infections | A04 |
| Other bacterial foodborne intoxications, NEC | A05 |
| Viral and other specified intestinal infections | A08 |
| *Liver infections* | |
| Acute hepatitis A | B15 |
| Acute hepatitis B | B16 |
| Other acute viral hepatitis | B17 |
| Chronic viral hepatitis | B18 |
| Unspecified viral hepatitis | B19 |
| *Respiratory infections* | |
| Respiratory tuberculosis, bacteriologically and histologically confirmed | A15 |
| Respiratory tuberculosis, not confirmed bacteriologically or histologically | A16 |
| Diphtheria | A36 |
| Whooping cough | A37 |
| Scarlet fever | A38 |
| Acute nasopharyngitis (common cold) | J00 |
| Acute sinusitis | J01 |
| Acute pharyngitis | J02 |
| Acute tonsillitis | J03 |
| Acute laryngitis and tracheitis | J04 |
| Acute obstructive laryngitis (croup) and epiglottitis | J05 |
| Acute upper respiratory infections of multiple and unspecified sites | J06 |
| Influenza due to certain identified influenza viruses | J09 |
| Influenza due to other identified influenza virus | J10 |
| Influenza due to unidentified influenza virus | J11 |
| Viral pneumonia, NEC | J12 |
| Pneumonia due to Streptococcus pneumoniae | J13 |
| Pneumonia due to Haemophilus influenzae | J14 |
| Bacterial pneumonia, NEC | J15 |
| Pneumonia due to other infectious organisms, NEC | J16 |
| Pneumonia in diseases classified elsewhere | J17 |
| Pneumonia, organism unspecified | J18 |
| Acute bronchitis | J20 |
| Acute bronchiolitis | J21 |
| Unspecified acute lower respiratory infection | J22 |
| *Sepsis* | |
| Streptococcal sepsis | A40 |
| Other sepsis | A41 |
| *Skin infections* | |
| Erysipelas | A46 |
| Herpesviral [herpes simplex] infections | B00 |
| Varicella (chickenpox) | B01 |
| Zoster (herpes zoster) | B02 |
| Smallpox | B03 |
| Monkeypox | B04 |
| Measles | B05 |
| Rubella | B06 |
| Viral warts | B07 |
| Other viral infections characterized by skin and mucous membrane lesions, NEC | B08 |
| Unspecified viral infection characterized by skin and mucous membrane lesions | B09 |
| Staphylococcal scalded skin syndrome | L00 |
| Impetigo | L01 |
| Cutaneous abscess, furuncle, and carbuncle | L02 |
| Cellulitis | L03 |
| Acute lymphadenitis | L04 |
| Pilonidal cyst | L05 |
| Other locations of skin and subcutaneous tissue | L08 |
| *Urogenital infections* | |
| Acute cystitis | N30.0 |
| Urinary tract infection, site not specified | N39.0 |
| Acute prostatitis | N41.0 |
| Chronic prostatitis | N41.1 |
| Inflammatory disease of uterus, except cervix | N71 |
| Inflammatory disease of cervix uteri | N72 |
| *Other infections* | |
| Tuberculosis of other organs | A18 |
| Miliary tuberculosis | A19 |
| Infection due to other mycobacteria | A31 |
| Listeriosis | A32 |
| Meningococcal infection | A39 |
| Actinomycosis | A42 |
| Nocardiosis | A43 |
| Bartonellosis | A44 |
| Other bacterial diseases, NEC | A48 |
| Bacterial infection of unspecified site | A49 |
| Cytomegaloviral disease | B25 |
| Mumps | B26 |
| Infectious mononucleosis | B27 |
| Viral conjunctivitis | B30 |
| Other viral diseases, not elsewhere classified | B33 |
| Viral infection of unspecified site | B34 |
| Streptococcus and staphylococcus as the cause of diseases classified to other chapters | B95 |
| Other specified bacterial agents as the cause of diseases classified to other chapters | B96 |
| Viral agents as the cause of diseases classified to other chapters | B97 |
| Other specified infectious agents as the cause of diseases classified to other chapters | B98 |
| Otitis externa in bacterial diseases classified elsewhere | H62.0 |
| Otitis externa in viral diseases classified elsewhere | H62.1 |
| Otitis media in bacterial diseases classified elsewhere | H67.0 |
| Otitis media in viral diseases classified elsewhere | H67.1 |
| Pyogenic arthritis | M00 |
| Meningococcal arthritis | M01.0 |
| Tuberculous arthritis | M01.1 |
| Arthritis in Lyme disease | M01.2 |
| Arthritis in other bacterial diseases classified elsewhere | M01.3 |
| Rubella arthritis | M01.4 |
| Arthritis in other viral diseases classified elsewhere | M01.5 |
| Inflammatory disorders of breast (mastitis, carbuncle, abscess) | N61 |

| Table S2. List of viral infections by ICD-10 code | |
| --- | --- |
| **Infection type** | **ICD-10 code** |
| Acute poliomyelitis | A80 |
| Atypical virus infections of central nervous system | A81 |
| Other viral encephalitis, not elsewhere classified (NEC) | A85 |
| Unspecified viral encephalitis | A86 |
| Viral meningitis | A87 |
| Other viral infections of central nervous system, NEC | A88 |
| Unspecified viral infection of central nervous system | A89 |
| Herpesviral meningitis | B00.3 |
| Herpesviral encephalitis | B00.4 |
| Varicella meningitis | B01.0 |
| Varicella encephalitis, myelitis and encephalomyelitis | B01.1 |
| Zoster encephalitis | B02.0 |
| Zoster meningitis | B02.1 |
| Zoster with other nervous system involvement | B02.2 |
| Measles complicated by encephalitis | B05.0 |
| Measles complicated by meningitis | B05.1 |
| Rubella with neurological complications | B06.0 |
| Mumps meningitis | B26.1 |
| Mumps encephalitis | B26.2 |
| Meningitis in viral diseases classified elsewhere | G02.0 |
| Encephalitis, myelitis, and encephalomyelitis in viral diseases classified elsewhere | G05.1 |
| Viral and other specified intestinal infections | A08 |
| Acute hepatitis A | B15 |
| Acute hepatitis B | B16 |
| Other acute viral hepatitis | B17 |
| Chronic viral hepatitis | B18 |
| Unspecified viral hepatitis | B19 |
| Acute nasopharyngitis (common cold) | J00 |
| Acute sinusitis | J01 |
| Acute pharyngitis | J02 |
| Acute tonsillitis | J03 |
| Acute laryngitis and tracheitis | J04 |
| Acute obstructive laryngitis (croup) and epiglottitis | J05 |
| Acute upper respiratory infections of multiple and unspecified sites | J06 |
| Influenza due to certain identified influenza viruses | J09 |
| Influenza due to other identified influenza virus | J10 |
| Influenza due to unidentified influenza virus | J11 |
| Viral pneumonia, NEC | J12 |
| Acute bronchitis | J20 |
| Acute bronchiolitis | J21 |
| Unspecified acute lower respiratory infection | J22 |
| Other sepsis | A41 |
| Herpesviral [herpes simplex] infections | B00 |
| Varicella (chickenpox) | B01 |
| Zoster (herpes zoster) | B02 |
| Smallpox | B03 |
| Monkeypox | B04 |
| Measles | B05 |
| Rubella | B06 |
| Viral warts | B07 |
| Other viral infections characterized by skin and mucous membrane lesions, NEC | B08 |
| Unspecified viral infection characterized by skin and mucous membrane lesions | B09 |
| Cytomegaloviral disease | B25 |
| Mumps | B26 |
| Infectious mononucleosis | B27 |
| Viral conjunctivitis | B30 |
| Other viral diseases, not elsewhere classified | B33 |
| Viral infection of unspecified site | B34 |
| Viral agents as the cause of diseases classified to other chapters | B97 |
| Otitis externa in viral diseases classified elsewhere | H62.1 |
| Otitis media in viral diseases classified elsewhere | H67.1 |
| Pyogenic arthritis | M00 |
| Rubella arthritis | M01.4 |
| Arthritis in other viral diseases classified elsewhere | M01.5 |

| Table S3. List of bacterial infections by ICD-10 code | |
| --- | --- |
| **Infection type** | **ICD-10 code** |
| Tuberculous meningitis | A17 |
| Bacterial meningitis, not elsewhere classified | G00 |
| Meningitis in bacterial diseases classified elsewhere | G01 |
| Bacterial meningoencephalitis and meningomyelitis, NEC | G04.2 |
| Encephalitis, myelitis, and encephalomyelitis in bacterial diseases classified elsewhere | G05.0 |
| Cholera | A00 |
| Typhoid and paratyphoid fevers | A01 |
| Other salmonella infections | A02 |
| Shigellosis | A03 |
| Other bacterial intestinal infections | A04 |
| Other bacterial foodborne intoxications, NEC | A05 |
| Respiratory tuberculosis, bacteriologically and histologically confirmed | A15 |
| Respiratory tuberculosis, not confirmed bacteriologically or histologically | A16 |
| Diphtheria | A36 |
| Whooping cough | A37 |
| Scarlet fever | A38 |
| Pneumonia due to Streptococcus pneumoniae | J13 |
| Pneumonia due to Haemophilus influenzae | J14 |
| Bacterial pneumonia, NEC | J15 |
| Streptococcal sepsis | A40 |
| Other sepsis | A41 |
| Erysipelas | A46 |
| Staphylococcal scalded skin syndrome | L00 |
| Impetigo | L01 |
| Cutaneous abscess, furuncle, and carbuncle | L02 |
| Cellulitis | L03 |
| Pilonidal cyst | L05 |
| Other locations of skin and subcutaneous tissue | L08 |
| Acute cystitis | N30.0 |
| Urinary tract infection, site not specified | N39.0 |
| Acute prostatitis | N41.0 |
| Chronic prostatitis | N41.1 |
| Inflammatory disease of uterus, except cervix | N71 |
| Inflammatory disease of cervix uteri | N72 |
| Tuberculosis of other organs | A18 |
| Miliary tuberculosis | A19 |
| Infection due to other mycobacteria | A31 |
| Listeriosis | A32 |
| Meningococcal infection | A39 |
| Actinomycosis | A42 |
| Nocardiosis | A43 |
| Bartonellosis | A44 |
| Other bacterial diseases, NEC | A48 |
| Bacterial infection of unspecified site | A49 |
| Streptococcus and staphylococcus as the cause of diseases classified to other chapters | B95 |
| Other specified bacterial agents as the cause of diseases classified to other chapters | B96 |
| Otitis externa in bacterial diseases classified elsewhere | H62.0 |
| Otitis media in bacterial diseases classified elsewhere | H67.0 |
| Pyogenic arthritis | M00 |
| Meningococcal arthritis | M01.0 |
| Tuberculous arthritis | M01.1 |
| Arthritis in Lyme disease | M01.2 |
| Arthritis in other bacterial diseases classified elsewhere | M01.3 |
| Inflammatory disorders of breast (mastitis, carbuncle, abscess) | N61 |

| Table S4. Competing risk analysis examining prospective associations between depression and hospitalisation for infection, with death for reasons other than infectious disease included as the competing outcome | | | |
| --- | --- | --- | --- |
|  | Fully adjusted* | |  |
|  | *HR (95% CI)* | *p value* |  |
| Any infection | 1.20 (1.15 to 1.26) | **<0.001** |  |
| Infection subtype |  |  |  |
| *CNS* | 0.97 (0.64 to 1.46) | 0.890 |  |
| *GI* | 1.44 (1.24 to 1.67) | **<0.001** |  |
| *Liver* | 1.83 (1.21 to 2.78) | **0.005** |  |
| *Respiratory* | 1.29 (1.21 to 1.38) | **<0.001** |  |
| *Sepsis* | 1.25 (1.05 to 1.50) | **0.014** |  |
| *Skin* | 1.05 (0.97 to 1.14) | 0.256 |  |
| *Urogenital* | 1.23 (1.14 to 1.34) | **<0.001** |  |
| *Other* | 1.23 (1.05 to 1.44) | **0.009** |  |
| Viral infection | 1.29 (1.19 to 1.39) | **<0.001** |  |
| Bacterial infection | 1.14 (1.08 to 1.21) | **<0.001** |  |
| CNS=Central nervous system; GI=Gastrointestinal  *Covariates: Age, sex, social deprivation, BMI, smoking status, frequency of alcohol intake, number of long-term physical conditions, CRP level. ALT level was also adjusted for in the analysis where hospital admissions for liver infection were the outcome. | | | |

| Table S5. Prospective associations between depression and hospitalisation for infection occurring 12 months after the baseline assessment | | | |
| --- | --- | --- | --- |
|  | Fully adjusted* | |  |
|  | *HR (95% CI)* | *p value* |  |
| Any infection | 1.19 (1.15 to 1.24) | **<0.001** |  |
| Infection subtype |  |  |  |
| *CNS* | 0.96 (0.65 to 1.40) | 0.820 |  |
| *GI* | 1.39 (1.20 to 1.62) | **<0.001** |  |
| *Liver* | 1.97 (1.24 to 3.12) | **0.004** |  |
| *Respiratory* | 1.27 (1.20 to 1.35( | **<0.001** |  |
| *Sepsis* | 1.18 (1.03 to 1.35) | **0.017** |  |
| *Skin* | 1.01 (0.93 to 1.10) | 0.811 |  |
| *Urogenital* | 1.28 (1.19 to 1.39) | **<0.001** |  |
| *Other* | 1.17 (1.00 to 1.37) | **0.055** |  |
| Viral infection | 1.25 (1.16 to 1.34) | **<0.001** |  |
| Bacterial infection | 1.16 (1.10 to 1.22) | **<0.001** |  |
| CNS=Central nervous system; GI=Gastrointestinal  *Covariates: Age, sex, social deprivation, BMI, smoking status, frequency of alcohol intake, number of long-term physical conditions, CRP level. ALT level was also adjusted for in the analysis where hospital admissions for liver infection were the outcome. | | | |

| Table S6. Prospective associations between depression and hospitalisation for infection in unimputed data | | | |
| --- | --- | --- | --- |
|  | Fully adjusted* | |  |
|  | *HR (95% CI)* | *p value* |  |
| Any infection | 1.19 (1.14 to 1.24) | **<0.001** |  |
| Infection subtype |  |  |  |
| *CNS* | 1.01 (0.69 to 1.47) | 0.963 |  |
| *GI* | 1.41 (1.22 to 1.64) | **<0.001** |  |
| *Liver* | 1.88 (1.21 to 2.93) | **0.005** |  |
| *Respiratory* | 1.26 (1.18 to 1.33) | **<0.001** |  |
| *Sepsis* | 1.14 (0.99 to 1.31) | **0.069** |  |
| *Skin* | 1.05 (0.97 to 1.14) | 0.219 |  |
| *Urogenital* | 1.23 (1.14 to 1.33) | **<0.001** |  |
| *Other* | 1.13 (0.96 to 1.32) | **0.141** |  |
| Viral infection | 1.26 (1.17 to 1.36) | **<0.001** |  |
| Bacterial infection | 1.14 (1.08 to 1.20) | **<0.001** |  |
| CNS=Central nervous system; GI=Gastrointestinal  *Covariates: Age, sex, social deprivation, BMI, smoking status, frequency of alcohol intake, number of long-term physical conditions, CRP level. ALT level was also adjusted for in the analysis where hospital admissions for liver infection were the outcome. | | | |

| Table S7. Logistic regression models examining prospective associations between depression and hospitalisation for infection | | | |
| --- | --- | --- | --- |
|  | Fully adjusted* | |  |
|  | *OR (95% CI)* | *p value* |  |
| Any infection | 1.22 (1.17 to 1.26) | **<0.001** |  |
| Infection subtype |  |  |  |
| *CNS* | 0.95 (0.66 to 1.36) | 0.795 |  |
| *GI* | 1.41 (1.22 to 1.61) | **<0.001** |  |
| *Liver* | 1.75 (1.17 to 2.62) | **0.006** |  |
| *Respiratory* | 1.30 (1.22 to 1.37) | **<0.001** |  |
| *Sepsis* | 1.19 (1.05 to 1.35) | **0.008** |  |
| *Skin* | 1.04 (0.96 to 1.13) | 0.293 |  |
| *Urogenital* | 1.26 (1.17 to 1.36) | **<0.001** |  |
| *Other* | 1.19 (1.02 to 1.38) | **0.024** |  |
| Viral infection | 1.27 (1.19 to 1.36) | **<0.001** |  |
| Bacterial infection | 1.16 (1.10 to 1.22) | **<0.001** |  |
| CNS=Central nervous system; GI=Gastrointestinal  *Covariates: Age, sex, social deprivation, BMI, smoking status, frequency of alcohol intake, number of long-term physical conditions, CRP level. ALT level was also adjusted for in the analysis where hospital admissions for liver infection were the outcome. | | | |
